# Patients’, clinicians’ and developers’ perspectives and experiences of artificial intelligence in cardiac healthcare: A qualitative study

**DOI:** 10.1177/20552076251328578

**Published:** 2025-06-16

**Authors:** Lesley Baillie, Adele Stewart-Lord, Nicola Thomas, Dan Frings

**Affiliations:** 1 College of Health and Life Sciences, London South Bank University, London, UK

**Keywords:** Artificial intelligence, patients, clinicians, stress echocardiography, perspectives, experiences

## Abstract

**Objective:**

This study investigated perspectives and experiences of artificial intelligence (AI) developers, clinicians and patients about the use of AI-based software in cardiac healthcare.

**Methods:**

A qualitative study took place at two hospitals in England that had trialled AI-based software use in stress echocardiography, a scan that uses ultrasound to assess heart function. Semi-structured interviews were conducted with: patients (*n = *9), clinicians (*n = *16) and AI software developers (*n = *5). Data were analysed using thematic analysis.

**Results:**

Potential benefits identified were increasing consistency and reliability through reducing human error, and greater efficiency. Concerns included over-reliance on the AI technology, and data security. Participants discussed the need for human input and empathy within healthcare, transparency about AI use, and issues around trusting AI. Participants considered AI's role as assisting diagnosis but not replacing clinician involvement. Clinicians and patients emphasised holistic diagnosis that involves more than the scan. Clinicians considered their diagnostic ability as superior and discrepancies were managed in line with clinicians’ diagnoses rather than AI reports. The practicalities of using the AI software concerned image acquisition to meet AI processing requirements and workflow integration.

**Conclusions:**

There was positivity towards AI use, but the AI software was considered an adjunct to clinicians rather than replacing their input. Clinicians’ experiences were that their diagnostic ability remained superior to the AI, and acquiring images acceptable to AI was sometimes problematic. Despite hopes for increased efficiency through AI use, clinicians struggled to identify fit with clinical workflow to bring benefit.

## Introduction

Artificial intelligence (AI) can be defined as: ‘the ability of computer systems to perform tasks that would usually require human levels of intelligence’.^1^ The World Health Organisation (WHO)^2^ identified the increasing role of AI in healthcare globally and the potential positive contribution. Whilst there is an emerging international body of literature reporting on views about AI use from clinicians and the public, fewer studies include perspectives and experiences from participants exposed to AI use in healthcare. This paper reports on a qualitative study that investigated AI developers’, clinicians’ and patients’ perspectives and experiences of AI software for interpreting images from stress echocardiography.

Stress echocardiography is a widely used investigation for the diagnosis of coronary artery disease. Despite guidelines, interpretation of the images can be subjective and operator-dependent.^1^ AI-based software showed potential for automatically processing stress echocardiography images, while reducing operator variability and increasing clinicians’ diagnostic confidence and accuracy.^3^ The AI software was developed through machine learning and is being tested in a Phase 3 clinical trial (‘Proteus’)^4,5^ and Phase 4 evaluations.^6^

Increased integration of AI into global healthcare systems appears inevitable as healthcare costs rise with ageing populations.^7^ Internationally, studies have investigated clinicians’ views about AI use in healthcare in hospitals^8–10^ and in primary care.^11,12^ Studies of the public's views have also been conducted.^13–15^ A systematic review of healthcare professionals’, patients’ and the public's views about AI in healthcare found many areas of agreement between them.^16^ Positive attitudes and openness towards AI use in healthcare have been reported from clinicians^11,17^ and the public,^18,19^ though with caveats and reservations. One study's participants believed that patients were unconcerned about AI use, trusting clinicians to make decisions about their care; however, patients’ views were not directly elicited.^17^

Suggestions for potential benefits of AI use in healthcare include earlier and quicker diagnosis^18,20^ and improved diagnostic accuracy with reduced human error.^17^ Increased efficiencies through use of AI have been proposed as a potential benefit.^11,18,20^ However, AI can provoke ethical and legal problems in healthcare.^9,21^ Data security concerns have been raised in some studies^17,18^ and the need for AI validation, transparency and explainability has been highlighted.^16^ The WHO^2^ identified the need to address ethical concerns and governance and has produced guidance for AI use in healthcare. Concerns about algorithm accuracy and possible bias of AI have also been raised^11,22^ with trust and confidence considered a crucial factor in acceptability.^17,18,23^ Healthcare professionals, patients and the public questioned who should be held accountable if adverse events arise from using AI.^16^ Some clinicians have emphasised that AI can only assist decision making, as clinicians remain legally responsible for clinical decisions.^24^ Others expressed scepticism about AI replacing human decision-making, which applies human judgment, use of clinical reasoning and values-based care.^12^ In some studies, clinicians asserted that AI cannot communicate health information sensitively and show empathy,^12,16,17^ and accordingly, patients reported concerns about diagnoses being conveyed by AI rather than humans.^25^

A systematic review^16^ of attitudes towards AI use in healthcare amongst healthcare professionals, patients and the public found there had been increasing numbers of studies between 2017 and 2021. However, only 10% of studies included participants with lived experience with AI tools.^16^ Therefore, Vo et al.^16^ considered it critical to assess participants’ attitudes towards their real-world use and exposure to AI applications. Whilst few studies have focussed on perspectives of AI use in stress echocardiography specifically, in one small qualitative study views were elicited through interviews with AI developers, IT staff and clinicians about the AI software.^17^ Outside this work, few studies have investigated perspectives of clinicians who have actual exposure to AI in practice, and even less involve patients who have been exposed to AI use in their healthcare. This study addressed this gap and was conducted to gain understanding of different perspectives and experiences of AI software. This is important as these may affect acceptability, future adoption and sustainability in practice. The study's aim was to investigate perspectives and experiences of AI developers, clinicians and patients about the use of AI-based software in cardiac healthcare. As such, it is one of the first to investigate the perspectives of clinicians and patients with actual experience of AI in healthcare, triangulated with the perspectives of the AI software developers.

## Methods

This qualitative study was part of a larger project^6^ which used multiple methods to evaluate the use of AI software developed to interpret images obtained during stress echocardiography. COREQ guidelines^26^ were used in preparation of this report.

### Data collection sites and participants

Semi-structured interviews (*n = *30) were conducted from May to December 2023 with AI developers, and clinicians and patients at two National Health Service (NHS) sites in England that had trialled an AI software product (EchoGo Pro) in stress echocardiography. Table 1 summarises the study sites, participants and interviews conducted. The AI developers’ interviews were longer as they discussed implementation extensively; these data will be reported in a separate paper focused on implementation of AI software. The aim was to recruit a range of individuals from these sites who could provide different perspectives. Recruitment continued until there were no new emerging themes.

**Table 1.** Sites, participants and interviews.

Site	AI software involvement	Participants	Number	Interview length
AI software company	Developed and trialled the AI software	Chief operating officer (*n = *1); director of research operations (*n = *1); project manager (*n = *1); services director (*n = *1); cardiac physiologist (research and education role) (*n = *1)	*N = *5	40–53 min (*M = *45 min)
Site 1: NHS Trust in the Midlands	Pilot Phase 4 trial of the AI software	Cardiac physiologists (*n = *2); echocardiographer (*n = *1); medical consultant (*n = *1); associate practitioner in cardiology (*n = *1)	*N = *5	27–41 min (*M* =* *33 min)
		Patient (male) who had undergone a stress echo test	*N = *1	23 min
Site 2: NHS Trust in the north of England	Phase 3 trial of the AI software	Consultant cardiologists (*n = *2); cardiac physiologists (*n = *3), specialist cardiac nurses (*n = *3), research nurses/practitioners who worked on the AI software trial (*n = *3)	*N = *11	17–38 min (*M* =* *27 min)
		Patients (female: *n = *3; male: *n = *5) who had undergone a stress echo test and took part in the Phase 3 trial	*N = *8	17–35 min (*M* =* *24 min)

### Ethical approval and patient and public involvement (PPI)

The study was approved by an NHS Research Ethics committee (IRAS 315284) and London South Bank University (ETH2223-0164). All participants were provided with participant information sheets and signed written consent forms prior to the interviews. Data were anonymised and stored securely. The project's Patient and Public Involvement (PPI) group, who comprised a diverse group of people with cardiac disease (*n = *10), met regularly and assisted development of the study documentation and reviewed recruitment plans, study progress and findings.

### Recruitment and data collection

Purposive sampling was used and one interview only was conducted with each participant. For each group of participants, a gatekeeper at that site approached individuals who met the eligibility criteria with an invitation to take part and a participant information sheet. If interested in participating, the individuals contacted the researchers, who responded by email, introducing themselves and their backgrounds, which were also explained in the participant information sheets. Numbers of participants approached but not willing to participate were not recorded, to maintain confidentiality with employers and the Trusts about who participated, which was an ethical requirement. The AI developers approached were those involved with the development and implementation of the software. At the NHS sites, research nurses approached clinicians who had exposure to the AI software and involvement with stress echo tests. For patient recruitment, at Site 1, a research nurse approached patients at stress echocardiography clinics. As the Phase 4 trial had completed some months previously, there was limited interest and only one patient (male), who had a stress echo test, was recruited. At Site 2, the Phase 3 trial was still in progress with follow-ups of participating patients. Research nurses approached a range of these patients and *n = *8 participants were recruited. Patients were given small gift vouchers to thank them for their time in taking part. Clinicians were also given small gift vouchers as the interviews were additional to their workload.

Each interview was conducted by one of two experienced qualitative researchers (LB: 25 interviews; AS-L: 5 interviews) who liaised throughout data collection. The researchers were female university academics with PhDs and healthcare backgrounds, and experience in evaluating service developments. They had had no involvement or experience with the AI software. The researchers kept field notes and reflective notes throughout data collection and analysis, striving to be reflexive and aware of any influence of their previous personal and professional experiences. The researchers used semi-structured interview guides (see Supplementary Files), developed by the project team and PPI group, comprising open questions and probes to clarify and seek greater depth.^6^ To promote consistency, the interviewers discussed the topic guides prior to starting the interviews, and critically reviewed the interview guides together after the first interviews with each participant group. All staff interviews (AI developers and clinicians) were conducted on MS Teams. Due to time constraints, there was no previous relationship established but the researchers established rapport at the start of the interviews. Patients had the option of telephone or MS Teams interviews but all selected telephone. As the interviews were not in-person, there could have been other people in their vicinity but this was beyond the researchers’ control. The interviews were audio-recorded with consent, professionally transcribed and then checked against the audio-recordings.

## Data analysis

The data were analysed using Braun and Clarke's reflective thematic analysis approach.^27^ No qualitative data analysis software was used but Microsoft Word functionality assisted the process. Table 2 summarises the six phases. These were used iteratively alongside the data collection. Both researchers worked on familiarization, coding and theme development in constant liaison. The early findings were presented to the staff groups who confirmed resonance. It was not possible to present the findings to the patients due to lack of ethical permission to re-contact them, taking into account their potentially precarious health status.

**Table 2.** Thematic data analysis phases and study application.^27^

Phase	Application
1. Familiarising yourself with the dataset	The researchers immersed themselves in the data through reading the transcripts and listening to the audio recordings.
2. Coding	Data were systematically coded, line by line, using inductive codes derived from the data, to capture meaning and concepts. New codes continued to be added throughout the coding process, which went through several rounds. The whole data set was reviewed in line with the final set of codes. Data segments for each code were compiled.
3. Generating initial themes	Codes with similar meanings/concepts were clustered and then potential themes identified, with relevant coded data collated for each candidate theme.
4. Developing and reviewing themes	The dataset and coded data extracts were reviewed against the candidate themes and reviewed for fit. The core focus, scope and viability of each theme and sub-theme was reviewed and relationships between the themes was considered
5. Refining, defining and naming themes	The themes were further reviewed to ensure each was clearly demarcated with a strong core concept. Informative names for the themes and sub-themes were developed
6. Writing up	The themes and sub-themes were written up, with data extracts for illustration.

## Results

Table 3 presents the major themes and sub-themes, which illustrate the nuances and diversity within the themes.

**Table 3.** Themes and sub-themes.

Themes	Sub-themes
Potential benefits of AI in healthcare	Increasing consistency and reliability Increasing efficiency
Concerns and caveats about AI use in healthcare	Over-reliance on AI technology Data security issues Need for human input and empathy Transparency about AI use Trust issues with AI
Role of AI in stress echocardiography diagnosis and limitations	How AI could contribute to diagnosis Diagnosis is holistic: more than the scan Clinicians’ superior diagnostic ability and managing discrepancies
Practicalities of using AI technology in stress echocardiography	Image acquisition to meet AI requirements AI fit with workflow

These themes and sub-themes are next discussed, with data extracts for illustration, identified in this way: AI1-5: AI developers

S1_C1-C5: Site 1 clinicians

S2_C1-C11: Site 2 clinicians

S1_P1: Site 1 patient

S2_P1-P8: Site 2 patients

### Theme 1 Potential benefits of AI in healthcare

Participants expressed positivity towards AI-based technologies, identifying potential benefits as increasing consistency, reliability and efficiency.

#### Increasing consistency and reliability

Participants considered that AI could increase consistency rather than relying on subjective diagnoses from individual clinicians. Work pressure, tiredness, lack of training and assumptions were perceived to lead to human error, affecting ability to diagnose consistently and accurately. Patients considered that AI technologies might reduce mistakes: ‘it does take out that element of distraction, tiredness that humans have, but machinery, technology, computers don’t’ (S2_P3). Clinicians identified that subjectivity and circumstances could affect human interpretation:

If you’re looking at bad left ventricular function all day, your eye – so what you will think is normal – will change. And if you were looking at really, really good function – i.e. too good function really: hyperdynamic function – all day, your opinion of ‘normal’ would change again. So, obviously, a computer, it wouldn’t do that. (S2_C3)

An AI developer referred to AI ‘creating equality in terms of patient access to diagnosis’ (AI3) and similarly a patient hoped that AI use would promote wider exposure to expertise, as well as removing human factors that could affect performance:

Say you had the three top cardiologists who were the best in the country in terms of echocardiographs, if the three of them decided to help build the AI system to interpret the images then in essence you would have the ultimate machine which didn’t have the human fallibility of perhaps being tired or perhaps just having a bad day. (S2_P4)

#### Increasing efficiency

Participants considered the potential for AI technologies to increase efficiency, hoping that clinicians could: ‘deliver their roles more succinctly and more appropriately and timely as well’ (S1_C3)*.* Some participants suggested that increased efficiency could reduce unnecessary testing, improving patient experience and reducing costs. A patient explained how AI might increase efficiency:

If the task is interpreting varying images, given a set of parameters that it's been taught, then I can see how potentially that could be so much more efficient than the human. (S2_P4)

Some clinicians similarly identified potential for the AI technology, if accurate, to automate stress echo test analysis:

If it is proven to work, this is something that could really optimise throughput in that it would reduce the analysis time, it would reduce the reliance on there being a person who does the physical analysis. (S2_C1)

Accordingly, participants raised the potential for AI to offer solutions to NHS staff shortages. An AI developer recalled interest from NHS England about whether AI software use could help reduce waiting lists for stress echocardiography: ‘potentially getting through some of the huge backlog of patients’ (AI2). Patients recalled long waiting times for their stress echo tests; they emphasised the seriousness of heart disease, hoping that AI might reduce waiting times and associated anxiety:

I think hopefully with this it may help speed things and put people at ease. Unfortunately, it's not like you’re dealing with a sore tooth or a broken toe that you can carry on living with, at the end of the day when you are looking at someone's heart, there's a lot of anxiety that comes with it. (S2_P7)

### Theme 2 Concerns and caveats about AI use in healthcare

Concerns about AI use in healthcare included potential over-reliance and data security issues. Participants emphasised the need for continuing human input, transparency about AI use, and trust in AI technology.

#### Over-reliance on AI technology

Patients much more than other participants raised concerns about over-reliance on AI for diagnosis. There were perceived risks that clinicians might accept AI results without checks or questioning:

It would be easy after a while to move into thinking that the machine is correct, AI is correct and moving on from there, I think the challenge would be to always, always keep that open mind when you get results from AI, just to always check. (S2_P2)

A few patients raised whether the use of AI technology in diagnosis could lead to loss of diagnostic skills and potential adverse effects if the technology failed. Patients identified the risk of cyber-attacks, and they questioned whether there would be contingencies in place: ‘if it [AI] crashes, what happens then in the hospital world?’ (S2_P5). Patients considered that AI should be used alongside clinician expertise so there was not a total reliance on the AI: ‘Use the two together so should something happen, a doctor can still fall back on their skills and diagnose and evaluate on paper’ (S2_P7).

#### Data security issues

Several patients raised data security issues and AI developers acknowledged concerns in relation to the AI technology: ‘they’re worried about their information being used’ (AI4). Whilst Site 2 clinicians reported very few patients declined to take part in the Phase 3 trial, those patients who did had data security concerns: ‘They didn't want their information out there’ ‘is what one patient said to me’ (S2_C3). Patients were assured that their data would be anonymized, before being transferred externally for AI analysis, but clinicians found some patients remained concerned about their data leaving the NHS hospital. A few clinicians discussed similar concerns about external data sharing and whether the data could be completely anonymised:

We’re sending all this data externally: are there going to be any things that could put at risk our patient confidentiality? Or is that something that can be completely anonymous when we send it across? (S1_C4)

#### Need for human input and empathy

Patients identified that ‘human touch’ was important, particularly for older people who may be less familiar with technology: ‘Especially for elderly people, they just sort of need that comforting hand’ (S2_P5). Most patients described feeling anxious about the stress echo test and emphasised the importance of support from clinicians. Some patients identified shortcomings of technologies as opposed to humans, leading to concerns about AI use in healthcare:

A machine, no matter how clever it is, it doesn’t know the emotional personal feel of relating one-to-one, one person talking to another person, it will never know. (S2_P2)

Another patient expressed reservations about whether AI could convey empathy or respond to emotional impact, stressing that some diagnoses are: ‘quite damaging to somebody or potentially quite upsetting’ (S2_P4); they expressed concern about diagnoses being delivered by AI without humans involved. Some clinicians too had reservations about how far AI could go in healthcare to replace humans: ‘You still need that human contact in healthcare I feel. I personally can’t see it taking over the role of a doctor’ (S2_C9).

#### Transparency about AI use

Many participants considered that transparency about AI use was essential for maintaining patient trust. Some patients expressed that they wanted to understand the rationale for AI use and to be reassured that: ‘the technology was safe and it worked and it had been certified that this is the best way of doing this test’ (S2_P4). However, some participants believed that when AI was completely integrated into healthcare, patients would not need to be told about its use. A few clinicians expressed that patients trust health professionals to use appropriate technologies in their care and they are only concerned about their diagnosis: ‘they just know that the scan has been done and hopefully the scan is good’ (S1_C3). An AI developer highlighted the various sources that might support diagnosis but would not be disclosed routinely: ‘The patient doesn’t necessarily need to know that the doctor has asked for support from either a book or the internet or another technology’ (AI1). Some patients expressed similar views, pointing out that they were not informed about how other diagnostic tools worked, and they trusted clinicians to use appropriate tools:

As long as I know at the end of the day that I’ve been in and I get some feedback whether everything is good or bad, I’m not really worried about how that outcome was achieved. (S2_P7)

#### Trust issues with AI

A crucial factor affecting acceptability of the AI based software for stress echocardiography related to trust. A patient asserted: ‘I would say the biggest thing is learning to trust the system’ (S2_P7). An AI developer found that clinicians needed to better understand AI-based decisions, to engender trust. However, it was too resource-intensive to provide detailed information about the AI decision-making as: ‘That would be embedded deep in the AI […] we couldn’t do it for each and every one because it was very, very time consuming’ (AI3).

AI developers emphasised the substantial evidence base for the stress echocardiography AI-based software. However, some clinicians questioned whether the images used during development were everyday images, as they found it problematic to produce images acceptable to the AI software (see sub-theme: ‘Image acquisition to meet AI requirements’). Therefore, clinicians questioned how the underpinning evidence aligned with the ‘real world’ of stress echos: ‘What is the image quality like? Is it feasible in the real, live, clinical setting: in the real world setting?’ (S1_C4). Clinicians clarified what would be necessary to engender their trust in the AI technology:

I think, probably, until it's producing accurate results time after time: it's picking up things that we’ve not seen, or ‘Oh, yes, the reports match my reports to the echo report.’ So I think, probably trust: trusting it. (S2_C5)

### Theme 3 Role of AI in stress echocardiography diagnosis and limitations

Both clinicians and patients considered that diagnosis involves more than interpreting a scan. From their experiences, clinicians believed their diagnostic ability remained superior to AI and so managed patients in line with their interpretation, regardless of the AI report.

#### How AI could contribute to diagnosis

Clinicians’ experiences of the AI software were in clinical trials and so clinicians were still reviewing patients’ scans, as well as receiving AI-generated reports. Both AI developers and clinicians agreed that legally, the clinician was responsible for the diagnosis regardless of whether AI was used. The AI software was considered by the developers as: ‘*a decision support tool’* (AI4). Clinicians considered AI's role should be assistive, not clinician replacement: ‘I think it would have to work in conjunction with the current physiologists and cardiologists rather than taking over their role’ (S2_C9). AI developers emphasised the power of AI and believed that it could detect changes that clinicians could not: ‘it's almost like getting an expert helper’ (AI1). Some patients similarly believed that AI could exceed human capabilities and so it could be: ‘a prompt for a doctor to look closer’ (S2_P4). Both clinicians and patients considered that AI might confirm diagnosis or flag an abnormal echo leading to a further review: ‘clarification, second opinion, being sure, making sure that nothing's missed’ (S2_C10).

AI developers expressed that the AI software would offer reassurance to inexperienced clinicians, and thus reduce referrals to senior clinicians. However, some clinicians were concerned that inexperienced clinicians might accept the AI's results unquestioningly, rather than being an aide to diagnosis. They emphasised clinicians needed to use AI as: ‘a “something else to guide them” as opposed to -it's not the be all or end all’ (S1_C2). Patients emphasised the importance of clinical input into their diagnosis and considered that AI, like any technology, was assisting rather than replacing clinicians. They were aware that, as they were involved in a trial, the cardiologist would make the diagnosis, but one patient raised whether AI views should override the clinicians’ views in some situations:

What if the professional in front of you isn’t actually the best at their job? So should the AI be allowed to override the professional or should it be able to force a review by someone else? (S2_P1)

#### Diagnosis is holistic: More than the scan

Clinicians explained that there was a holistic approach to stress echocardiography diagnosis, considering the scan alongside the patient history and clinical observation during the stress echo test. They contrasted this approach with AI, which only used the image and measurements. There was concern that without considering these additional factors: ‘the AI report might under-estimate the risk in some cases’ (S2_C1). Similarly, a patient believed that the consultant would take other information about them into account too, rather than relying on the AI report alone:

If he has got the patient notes and then he's got the AI thing, he can at least see if it is reasonable for what his expectations were or whether it's throwing up something odd or something doubtful on the AI. (S2_P2)

Clinicians emphasised the importance of observing patients during echo stress tests and patients too referred to the presence of clinicians during their stress echo tests, including the cardiologist, and the support and monitoring they experienced.

#### Clinicians’ superior diagnostic ability and managing discrepancies

The clinicians reported that they could interpret images that would be rejected by the AI software, as: ‘a clinician may require less clarity, lower resolution pictures, and still make a clinical decision’ (S2_C4). Another clinician commented that until AI could read any scans, just like a human, the AI could not be relied on; there were currently images where: ‘The human eye could read it but the computer couldn’t’ (S2_C5). Where there were discrepancies, clinicians questioned the accuracy of the AI software, rather than their own interpretation, due to the newness of the technology:

If there is a bit of discrepancy, obviously, the first thing that many reporters questioned is, ‘Oh, is the AI actually accurate?’ as opposed to questioning our accuracy, which we’re more confident with. (S2_C1)

Clinicians had identified minor abnormalities and more major coronary artery disease from scans that were reported normal or low risk by AI. Where there were discrepancies, patients were treated in line with clinicians’ interpretations, leading to further investigations and treatments:

The patients were referred on for appropriate treatment, in one case, I think coronary angiography and then a coronary artery bypass graft. And, in another case, referred for coronary angiography and, I think, possibly stenting. […] obviously, as responsible clinicians, we had to carry on and make sure the patient got appropriate treatments. (S2_C2)

Whilst clinicians acknowledged that humans are not always right, their actual experiences with AI had not induced confidence:

We’re not infallible. But it turned out, on these occasions, that we were proved to be correct and then further tests showed that, actually, there was significant coronary artery disease. So, yes. So, straightaway, you think, ‘Well, if you can’t rely on the software’. (S2_C3)

Whilst some participants hypothesized that the AI software would help to alleviate errors and identify undetected abnormalities, there were no actual experiences of the AI software detecting disease missed by clinicians:

We’ve not had a single one which was the opposite: i.e. which showed we said it was normal but it [AI] said it was abnormal and then it turned out to be that [the AI] was right. (S2_S3)

Overall, clinicians’ diagnostic abilities were considered currently superior to the AI: ‘Our ability to differentiate remains higher, obviously: effectively, we’re the gold standard’ (S2_C2). Clinicians considered that humans should be able to accurately diagnose every patient: ‘We wouldn’t be doing our job if we weren’t able to diagnose every patient’ (S2_C9); they therefore expected that AI should perform to the same standard.

### Theme 4 Practicalities of using AI technology in stress echocardiography

Clinicians discussed their practical experiences of using the AI software from two key perspectives: image acquisition to meet AI requirements, and how AI software fitted with workflow.

#### Image acquisition to meet AI requirements

Clinicians recalled guidance from AI developers about image acquisition for the AI software, and some found that any problems decreased over time. However, other clinicians reported continuing difficulties with acquiring images that could be submitted to the AI software without rejection. A patient recalled the cardiologist reviewing her scans to determine eligibility for the trial: ‘whether or not the scans they took of my heart were suitable for use’ (S2_P3). Several patients recalled that contrast had to be used to improve the images, but clinicians received feedback that use of contrast increased potential rejection of images: ‘“Perhaps try and don’t use contrast whilst we’re taking the pictures if you can” because it seemed to accept more that weren’t using contrast’ (S2_C7).

As acceptability by the AI software depended on image quality, clinicians: ‘were only sending the clearest and best images to them because obviously, it wouldn’t be able to analyse the ones that are more unclear’ (S2_C8). Images that were perceived good enough for interpretation by clinicians could be rejected by AI:

There would be times when we felt we had good images and we’d done a good test but, when that data was uploaded, those images were rejected. The team felt a little disheartened. (S2_C11)

Obtaining images that AI could read was considered stressful and frustrating at times, as they were ‘trying to get the perfect images basically to be able to be analysed’ (S2_C8). It seemed the AI software could read straightforward and easily obtained images but images obtained from patients with more complex conditions were often not possible. Clinicians questioned the value of AI software that could not analyse the full range of images:

If the AI isn’t able to interpret images that are away from the norm, is that really something that is going to be used regularly or are we just waiting for that to improve? I am a bit sceptical, I would say, at the moment. (S2_C9)

Acquisition of images acceptable to the AI software took longer, particularly at the start, though some believed this would improve over time if AI software use became normalised. However, longer scanning times meant patients were kept on medication (dobutamine) for longer than usual, with a perceived higher risk of detrimental effects: ‘the longer it goes on, the more risk of having adverse effects to it’ (S2_C8). A clinician questioned whether any consideration had been given to this unintended effect and the potential risk to patients from being administered dobutamine for longer periods of time as: ‘I would say the effect is not significant but certainly not absent’ (S2_C4). Patients reported that dobutamine administration felt uncomfortable and expressed nervousness and sometimes fear: ‘I was worried that they would give me this medicine, maybe I could have a heart attack while I’m there’ (S2_P8).

#### AI fit with workflow

A Site 1 clinician reflected that trialling the AI software provided an opportunity to explore how AI could fit into stress echocardiography workflows: ‘How do we bring AI into clinical workflows as a more mainstream thing and what are the challenges associated with it?’ (S1_C3). Both AI developers and clinicians doubted whether AI use could reduce stress echo test appointment times:

Personally, I can’t see how it would reduce our workload here and now in our department simply because they’ll still be having the same test with us. (S1_C1)

A patient also recognised that regardless of whether AI was used to assist interpretation, there would still need to be clinicians to conduct the test: ‘you kind of have to have someone there, moving the sensor around to get the images’ (S2_P1).

The current time lag in the receipt of AI reports was considered a limitation. Clinicians believed there would be more potential benefit if the reports were received contemporaneously, rather than 20 minutes or more later:

If that's something [getting an AI report] that we can do it in conjunction, as we’re doing the scan, then that’d be great because you’re not causing any delay in time and, at the same time, we don’t have to do any post-processing at the end. (S1_C4)

Clinicians explained that their current stress echocardiography system is a ‘one-stop shop’ where patients are tested, reviewed and then informed of the diagnosis in the clinic. They could see little added benefit of using the AI software unless the time-lag was reduced, as waiting for the AI report before confirming diagnosis would impact on clinics. For example, patients would have to remain at the centre longer:

Unless you’re going to tell the patient to go and sit outside and wait half-an-hour and then come back again – which adds another layer of complexity to a clinic – by that time, most of them are on the way home in a car or on the bus. (S2_C2)

There was some variation amongst patients interviewed about when they had received their stress echo test results but most site 2 patients recalled being given the result straightaway, as clinicians had asserted, for example: ‘he [cardiologist] looked at it and said everything is fine’ (S2_P3).

Clinicians were aware that some stress echo centres had different systems for reporting scans. One clinician (S2_C2) suggested that use of AI might fit better at centres where a consultant cardiologist did batch reporting at the end of each day, reviewing each image alongside the patient's notes and the history taken by the physiologist during the test.

## Discussion

Few studies have explored and triangulated the multiple perspectives and experiences of the wide and diverse range of stakeholders associated with AI use in healthcare. We investigated perspectives and experiences of AI developers, clinicians and patients about the use of AI based software for interpreting images from stress echocardiography. Findings showed positivity towards AI use but clinicians suggested that their diagnostic ability remained superior to the AI and asserted that acquiring images acceptable to AI was sometimes problematic. Despite hopes for increased efficiency through AI use, clinicians struggled to identify fit with clinical workflow to bring benefit. These findings may resonate more widely around AI use in healthcare and are next discussed in the context of the literature.

There have been proposals that AI tools could guide clinicians in the decision-making process, with their output being integrated into the wider clinical picture.^1^ We found that participants viewed AI as an adjunct to diagnosis, but should not replace clinicians’ decision-making. Patients considered their cardiologists should check AI-generated results, believing that AI-generated results should not be accepted without clinician involvement. These views concur with previous study findings that ascertained clinicians’ views^17,21,28^ and those of patients and the public.^18,19,25,29^ Similarly, the WHO^2^ stresses that AI use should not undermine human autonomy and that humans should remain in control of health-care systems and medical decisions. We found that a few participants raised legal implications, pointing out that clinicians remained legally responsible for diagnostic decisions. It has been argued that a precondition for clinical use of AI is clarity about the legal liability for its usage.^8^

Participants considered that AI use could reduce subjectivity and increase accuracy and consistency in diagnosis. However, when clinicians found discrepancies between the AI software reports and their own interpretation, they concluded that the AI report was inaccurate, as they considered their ability as the ‘gold standard’ for making diagnoses. These perspectives from actual experiences using AI concur with views reported in other studies.^9,29^ The WHO^2^ asserted that the use of AI should not undermine human autonomy, and that in the context of health care, this means that humans should remain in control of health-care systems and medical decisions. Clinicians in our study acted in line with their diagnostic views, regardless of the AI reports, but one patient questioned whether this was always the right approach, referring to possible variation between clinicians’ performance. Whilst it has been suggested that AI could be particularly valuable for clinicians with less experience,^1^ we found that clinicians were concerned that less experienced clinicians might rely on the AI report, accepting it unquestioningly. Furthermore, both clinicians and patients emphasised that diagnosis involved more than the measurements and image analysis used for AI decision-making; it also involved consideration of the patient's history and the observations carried out during the stress echo test. The individuality of patients has been emphasised with doubts that AI could replace human judgment.^30,31^

Trust was found to be a central issue for the use of AI, which confirms results from other studies: with the public^13,15^ and clinicians.^11,32^ Accordingly, Gao^33^ identified that lack of trust in AI is a key reason behind negative attitudes toward medical AI. We found that clinicians emphasised the need for trust and confidence in the AI software; they questioned some of the AI decisions but could not access information as to how the AI software carried out the analysis. Whilst clinicians were aware that the AI software was developed based on thousands of images, they queried whether these images were representative of the ‘real world’ of images, including images of lower quality. In other studies, too, participants emphasised the importance of clinicians being confident in the evidence underpinning AI software.^17,20^ There have been public concerns about the evidence base for healthcare AI, with potential harm to patients if flawed datasets are used.^19^ In stress echocardiography, Fazakarley et al.^17^ suggested that trust in AI could be promoted by transparency about how the AI was developed, ensuring testing across diverse populations, and providing information about secure storage and management of patient data. The need for regulation and quality control has also been suggested for engendering trust in AI software.^17,20^

A key practical issue for clinicians concerned acquisition of images acceptable for the AI software. A systematic review of the use of AI in echocardiography identified that the quality of the AI model depends on the quality of the image acquisition.^34^ Clinicians in our study reported that the AI software could only interpret high quality and very clear images and they pointed out the variability between individuals, for example, body size; it seemed that AI struggled to read images of more complex patients. Some clinicians identified that acquiring images that could be read by the AI software took longer and accordingly, patients were administered dobutamine for longer, raising concerns about increased risk of adverse effects to patients. There have been suggestions that AI software could increase efficiency in healthcare,^12,35^ but impact is dependent on the AI tool fitting with clinicians’ workflow.^17^ We found that from practical experience with the AI software, clinicians expressed uncertainty about how clinician workload could be impacted due to the nature of stress echo tests, the current systems and workflows in place for processing the images, and delays in receiving the AI software reports.

Healthcare professionals have reported that patients have few concerns regarding AI software.^17^ However, some patients in our study identified the risk of over-reliance on AI software and they suggested the need for contingencies for technological failure. Concerns about having clinical systems that are highly dependent on AI, and what would happen should there be a system-wide failure, have been previously raised.^19^ We found some patients were concerned that over-reliance could lead to deskilling of clinicians, which supports concerns raised in other studies too.^17,18^ Whilst patients were reassured about data being anonymised, some concerns about data security remained, supporting other studies’ findings, within stress echocardiography specifically^17,34^ and more widely across healthcare.^16^ Following on from a UK-based study, there were suggestions for a regulatory framework for the handling of NHS data during AI use.^18^

Some patients and clinicians in our study were concerned that AI should not supersede human touch entirely, emphasising the importance of human interactions and empathy, which they doubted AI could deliver. These findings support those from other studies about perspectives of AI in healthcare.^12,15,16^ In an Australian study, the public were found to value human contact and discretion more than any speed, accuracy or convenience that AI could offer; they believed AI systems should augment rather than replace humans in health and social care.^15^ It has been asserted that the absence of the humanistic care factor is an important reason underpinning negativity towards medical AI.^33^ We found there were varying views about transparency of AI use with some participants feeling that transparency was an important principle, especially as the AI technology was new. In a previous study based in stress echocardiography, healthcare professionals raised transparency as important for acceptability of AI tools to clinicians and patients.^17^ Other studies have emphasised the need to inform patients about AI use,^29^ and a right for patients to choose whether an AI tool is used in their care and be able to opt-out of AI involvement if they feel strongly.^19^ However, some participants in our study believed that patients generally trust clinicians to use the most appropriate tools in their care, and this trust extends to AI use, with a belief that patients are not interested in the underlying technology of diagnostic tools.

Our study findings support and expand other conceptual accounts of AI uptake, such as Mayer's Organisational Model of Trust,^36^ which has been widely applied to AI.^37–39^ This model highlights the importance of trust in AI usage/acceptability – suggesting trust itself is predicted by perceived system ability, benevolence in design intent, and integrity (acceptable design principles). Our findings suggest challenges to factors such as benevolence, in that there are some concerns around unintended consequences, and system ability, both in terms of accuracy but also image requirements. There were varied views between different stakeholder groups, suggesting that approaches to AI acceptance need to be understood from a multi-stakeholder perspective. Our study findings highlight important implications for the acceptability and scalability of AI, suggesting that just focussing on a single stakeholder group could neglect key concerns of others. The findings also suggest ways to improve future AI design goals. One possibility is that AI may be more readily adopted where, rather than attempting to increase efficiency or make marginal outcome gains, it addresses important unmet needs. For instance, differentiating between disease sub-classifiers to allow for targeted treatment approaches may be seen as less possible by clinicians than AI. This design approach would require greater pre-development stakeholder involvement, but would likely increase perceptions of value added and likely efficacy of outcomes. Future work could also focus on identifying unmet needs

This study highlights the variation between developer expectations and clinician experience and future research could investigate how this developer-clinician gap could be bridged effectively to optimize potential benefits of AI software to healthcare. Future studies could further explore the role of AI in diagnosis, factors affecting clinicians’ trust in AI software and how AI software can be integrated into healthcare workflows without unintended consequences. Further research could also investigate the possible influence of participants’ demographic characteristics, and clinician roles, on experiences and perspectives of AI use in healthcare. Strengths of this study include that the clinicians and patients had had actual experience of AI software in practice; many previous studies have elicited views about AI rather than actual experiences. This study builds on a previous qualitative study of EchoGo Pro use,^17^ but included a wider sample of clinicians who had actually used the AI software in practice and patients who had been exposed to AI use. Limitations of the study include that data collection was from clinicians at only two NHS sites in one country (England) and there were small samples, though these elicited rich data. Participants willing to be interviewed may have had particular perspectives of AI software; other individuals could have different views. Site 2 patients had all been participants in the AI software Phase 3 trial; their perspectives may differ from patients without exposure to AI software in their care. The study was based on experiences of only one type of AI software in one clinical context; perspectives and experiences of AI software use may differ for other types of healthcare AI beyond the specific software studied. As a qualitative study, we did not intend results to be generalizable, but we have included contextual information about settings and the AI software to promote transferability where applicable.

## Conclusions

This study has uniquely brought together the experiences of AI developers, clinicians who used AI software in practice, and patients who had been exposed to AI software in their care. The findings support and expand existent models of AI adoption in healthcare. Patients and clinicians are generally open towards AI-based technologies in healthcare and, like AI developers, can identify potential benefits including greater consistency and efficiency. However, these potential benefits had not yet been experienced in relation to AI software for stress echocardiography and AI software was considered an adjunct to clinicians’ decision-making rather than replacing their input. This study illuminates patients’ concerns about data security, over-reliance on AI software and loss of human input in their care. Few studies have explored clinicians’ direct experience with AI software, and unique practical challenges were revealed, thus highlighting the importance of clinician early involvement in both initial exploration and subsequent development of AI technologies. Clinicians’ trust and confidence in AI is essential for acceptability but was not yet achieved. Clinicians’ experiences were that their diagnostic ability remained superior to the AI, and acquiring images acceptable to AI could be challenging. To maximise benefits of AI software in healthcare, it is essential to consider implications for use in practice, including potential adverse effects, the role of the software and fit with workflow.

## Supplemental Material

sj-docx-1-dhj-10.1177_20552076251328578 - Supplemental material for Patients’, clinicians’ and developers’ perspectives and experiences of artificial intelligence in cardiac healthcare: A qualitative study Supplemental material, sj-docx-1-dhj-10.1177_20552076251328578 for Patients’, clinicians’ and developers’ perspectives and experiences of artificial intelligence in cardiac healthcare: A qualitative study by Lesley Baillie, Adele Stewart-Lord, Nicola Thomas and Dan Frings in DIGITAL HEALTH

sj-docx-2-dhj-10.1177_20552076251328578 - Supplemental material for Patients’, clinicians’ and developers’ perspectives and experiences of artificial intelligence in cardiac healthcare: A qualitative study Supplemental material, sj-docx-2-dhj-10.1177_20552076251328578 for Patients’, clinicians’ and developers’ perspectives and experiences of artificial intelligence in cardiac healthcare: A qualitative study by Lesley Baillie, Adele Stewart-Lord, Nicola Thomas and Dan Frings in DIGITAL HEALTH

sj-docx-3-dhj-10.1177_20552076251328578 - Supplemental material for Patients’, clinicians’ and developers’ perspectives and experiences of artificial intelligence in cardiac healthcare: A qualitative study Supplemental material, sj-docx-3-dhj-10.1177_20552076251328578 for Patients’, clinicians’ and developers’ perspectives and experiences of artificial intelligence in cardiac healthcare: A qualitative study by Lesley Baillie, Adele Stewart-Lord, Nicola Thomas and Dan Frings in DIGITAL HEALTH

sj-pdf-4-dhj-10.1177_20552076251328578 - Supplemental material for Patients’, clinicians’ and developers’ perspectives and experiences of artificial intelligence in cardiac healthcare: A qualitative study Supplemental material, sj-pdf-4-dhj-10.1177_20552076251328578 for Patients’, clinicians’ and developers’ perspectives and experiences of artificial intelligence in cardiac healthcare: A qualitative study by Lesley Baillie, Adele Stewart-Lord, Nicola Thomas and Dan Frings in DIGITAL HEALTH
