# Supplementary material for: Patients’, clinicians’ and developers’ perspectives and experiences of artificial intelligence in cardiac healthcare: A qualitative study
Source: Digit Health. 2025 Jun 16;11:20552076251328578. doi: 10.1177/20552076251328578 (PMC12174740; doi:10.1177/20552076251328578)
Supplement: sj-docx-1-dhj-10.1177_20552076251328578 - Supplemental material for Patients’, clinicians’ and developers’ perspectives and experiences of artificial intelligence in cardiac healthcare: A qualitative study [file sj-docx-1-dhj-10.1177_20552076251328578.docx]

**interview guide: AI developers**

**Full title of Project:** Evaluating an AI driven stress echocardiography system (EASE)

IRAS Project ID: 315284

**What is the purpose of the study?**

This study aims to explore the acceptability, patterns of use, perceptions of

perceived safety of EchoGo Pro (a stress echocardiography system).

**The questions are there as a guide. The aim is that as the interviewee speaks, and the interviewer listens, areas are covered in a relaxed and flexible way. This will promote a semi- structured approach to the interview, rather than a structured interview, and provide opportunity for new areas to develop**

| **No** | **Question/Topic** | **Probes** |
| --- | --- | --- |
|  | **Introduction** | Remind participant about the aims and objectives of the research. Have extra copy of participant information sheet available  Check the participant is happy to have interview recorded  Answer any questions  Confirm consent form signed  Discuss confidentiality |
| **1** | **Can you tell me about your role within the AI development company?** | Tell us about the different activities you are involved in  What is your role in relation to EchoGo Pro? |
| **2** | **Tell me about your first encounter with EchoGo Pro** | When and where  How was EchoGo Pro introduced in the AI company?  What were your initial responses to the use of EcoGo Pro in practice? |
| **3** | **Thinking about the Implementation of EchoGo Pro at NHS sites:**  **Are you able to provide examples of sites where EchoGoPro was implemented and where not** | Was there high level support for implementation eg. Exec team, ICT etc? Explore response  What type of support would have helped?  Who led the implementation? Are they still using EchoGo Pro?  If not, probe why they are not using it  Were there new staff appointed to support the implementation? Explore response (roles, how they came about)  What type of staff support would have been helpful?  How were staff prepared to engage with NHS sites?  What resources and facilities were developed to support EchoGoPro implementation?  What support / resources should have been put in place to enable the implementation |
| **4** | **What are your views about EchoGo Pro and its perceived impact:** | What do you feel are the key elements of the EchoGoPro?  What are your views about how using EchoGoPro has impacted (or could impact) (probe for examples):   - patient/family experiences - staff experiences - workload - safety |
| **5** | **Could you tell me about any challenges you have faced implementing EchoGo Pro** | Explore the reasons for the challenges  How have you managed the challenges?  What are the perceived barriers to the use of EchoGo Pro?  What do you think are the enablers? |
| **6** | **How effective do you think EchoGo Pro is/could be in managing cardiovascular disease?** | Explore responses and the reasons |
| **7** | **What are the benefits of using EchoGoPro in your opinion?** | Explore the reasons for responses |
| **8** | **Have there been any changes or developments to how EchoGo Pro is used since first implemented?** | How did these happen and why?  What will ensure sustainability?  What are the specific resources required to ensure continuity? |
| **9** | **According to your role and interaction with clinical staff – how well is EchoGo Pro tolerated / accepted?** | Think about your interactions with clinical staff, what has been their response? Do you use the word AI when explaining the technology? What has been the response? |
| **10** | **Is there anything further that you want to tell me about EchoGo Pro** | Encourage reflection and learning from the experience |
|  | **Summarise**  **Thank participant** | Outline what happens next  Close |
