# Supplementary material for: Patients’, clinicians’ and developers’ perspectives and experiences of artificial intelligence in cardiac healthcare: A qualitative study
Source: Digit Health. 2025 Jun 16;11:20552076251328578. doi: 10.1177/20552076251328578 (PMC12174740; doi:10.1177/20552076251328578)
Supplement: sj-docx-2-dhj-10.1177_20552076251328578 - Supplemental material for Patients’, clinicians’ and developers’ perspectives and experiences of artificial intelligence in cardiac healthcare: A qualitative study [file sj-docx-2-dhj-10.1177_20552076251328578.docx]

**NHS clinicians: Interview guide**

**Full title of Project:** Evaluating an AI driven stress echocardiography system (EASE)

IRAS Project ID: 315284

**What is the purpose of the study?**

This study aims to explore the acceptability, patterns of use, perceptions,

perceived safety of EchoGo Pro (a stress echocardiography system).

**The questions are there as a guide. The aim is that as the interviewee speaks, and the interviewer listens, areas are covered in a relaxed and flexible way. This will promote a semi- structured approach to the interview, rather than a structured interview, and provide opportunity for new areas to develop**

| **No** | **Question/topic** | **Probes** |
| --- | --- | --- |
|  | **Introduction** | Remind participant about the aims and objectives of the research. Have extra copy of participant information sheet available  Check the participant is happy to have interview recorded  Answer any questions  Confirm consent form signed  Discuss confidentiality |
| **1** | **Can you tell me about your role as healthcare professional/clinician** | Tell me about the different activities you are involved in  What is your role in the cardiovascular disease patient pathway? |
| **2** | **When and where were you first made aware of EchoGoPro** | How did you learn about EchoGoPro and from who?  What were your initial responses to the use of EcoGoPro? |
| **3** | **Thinking about the implementation of EchoGoPro and support:** | What levels of support were available for implementation? Explore the individuals and levels of support (i.e. departmental, strategic, leadership)  What type of support would have helped at implementation?  Who led the implementation?  Are they still using EchoGoPro?  Were there new staff appointed to support the implementation? Explore response (roles, how they came about)  What type of staff support would have been helpful?  How were staff prepared?  What resources and facilities were developed to support EchoGoPro implementation?  What support / resources should have been put in place to enable the implementation? |
| **4** | **What are your views about EchoGoPro and impact:**  **What are your perceived views of AI use as part of a stress echo?** | What do you feel are the key elements of the EchoGoPro?  What are your views about using EchoGoPro and how this has impacted on (probe for examples):   - patient/family experiences - staff experiences - workload - safety |
| **5** | **Could you tell me about any challenges you have faced using / implementing EchoGoPro?** | Explore the reasons why  How have you managed the challenges?  What are the perceived barriers to the use of EchoGoPro?  In your experience what has affected the implementation in your NHS Trust?  What do you think are enablers? |
| **6** | **How effective do you think EchoGoPro is/could be in managing cardiovascular disease?** | Explore responses and reasons  Does it provide decision making support?  What are your experiences of the accuracy of results?  How do you as clinicians respond to any discrepant information from EchoGoPro reports? How does this influence your decision on the management of the patient?  How do you think EchoGo Pro could affect patient management? |
| **7** | **What are the potential benefits of using EchoGoPro in your opinion?** | Explore the reasons for responses |
| **8** | **Have there been any changes or developments to how EchoGoPro is used since first implemented?** | How did these happen and why?  What will affect sustainability?  What are the specific resources required to ensure continuity? |
| **9** | **According to your role and interaction with patients – how well is EchoGoPro tolerated / accepted?** | Think about your interactions with patients, what has been their response? Do you use the word AI when explaining the technology? What has been the response? |
| **10** | **Is there anything else that you want to tell me about EchoGoPro** | Encourage reflection and learning from the experience |
|  | **Summarise**  **Thank participant** | Outline what happens next |
