# Supplementary material for: Patients’, clinicians’ and developers’ perspectives and experiences of artificial intelligence in cardiac healthcare: A qualitative study
Source: Digit Health. 2025 Jun 16;11:20552076251328578. doi: 10.1177/20552076251328578 (PMC12174740; doi:10.1177/20552076251328578)
Supplement: sj-docx-3-dhj-10.1177_20552076251328578 - Supplemental material for Patients’, clinicians’ and developers’ perspectives and experiences of artificial intelligence in cardiac healthcare: A qualitative study [file sj-docx-3-dhj-10.1177_20552076251328578.docx]

**Interview guide: patients**

**Full title of Project:** Evaluating an AI driven stress echocardiography system (EASE)

IRAS Project ID: 315284

**The questions are there as a guide. The aim is that as the participants (patients) speak and the facilitator listens, the topic areas will be covered in a relaxed and flexible way. As patients’ experiences of the use of EchoGo Pro will vary, the interview guide will be used flexibly.**

|  | **Question** | **Probes** |
| --- | --- | --- |
|  | **Introduction** | Remind participants about the aims and objectives of the research. Have extra copy of participant information sheet available  Check the participant is happy to have interview recorded  Answer any questions  Confirm consent form signed  Discuss confidentiality and data privacy issues e.g., how their responses will be used/stored/anonymised and invite/address comments/concerns. |
| **1** | **Can you tell us a little bit about yourself and your experience of having a heart echo stress test.** | NB Clarify the difference between a standard ECG based stress test often done on a treadmill, and an echo stress test often done on a stationary bike or with medication |
| **2** | **Before we start, I am going to explain the technology (software) called EchoGo Pro (*explanation as needed, including artificial intelligence, depending on patient experience and recall*)** | The process of recording and interpreting scans.  Explanation of the technology in terms of how, why and when it is used (features)  Where the hospital is now in terms of using the technology (e.g. completing a trial) |
| **3** | **Do you recall being advised that EchoGo Pro will be used to assist with interpreting the results of your stress echocardiogram?** | If so, when and where?  How did you learn about EchoGo Pro and from who?  What were your initial responses to the use of EchoGo Pro? |
| **4** | **Thinking about your experiences of EchoGo Pro:** | How well was the use of the software explained to you?  Who explained the use of EchoGo Pro?  What were your views about the hospital using EchoGo Pro after the explanation - did you have any questions?  How prepared did you feel for your echo after the explanation?  Were there any adjustments needed for you to have your echo carried out? If yes, explore response  How were the results from your stress echo explained? Did they mention EchoGo Pro? |
| **5** | **What is your view of EchoGo Pro now?** | What do you feel is or could be the most important feature or benefits of the software? |
| **6** | **What are your views on using Artificial Intelligence in helping health care professionals make a diagnosis or decision about your care?** | What are your reasons for this view?  What do you think are the barriers and enablers to using AI in healthcare ie, what makes hospitals start or stop using AI?  Could you tell us about any concerns regarding the use of AI in healthcare.  Do you feel patients should be told if AI is being used in decision making about your treatment or management?  Have you any experience of AI being used in healthcare decision making? What was your view on this?  Have you used/experienced AI in any other setting? If yes, what are your views/experience about this? |
| **7** | **How comfortable would you feel knowing your doctor uses AI software to help their decision making?** | Explore the reasons for responses |
| **8** | **What are the possible benefits of using AI in health and social care generally (not just for heart scans) in your opinion?** | Explore the reasons for responses |
| **9** | **What are the possible challenges of using AI in health and social care generally (not just for heart scans) in your opinion?** | Explore the reasons for responses |
| **10** | **Is there anything else that you want to tell me about EchoGo Pro or AI that we have not discussed so far?** | Encourage reflection and learning from the experience |
|  | **Summarise**  **Thank participant** | Outline what happens next and a reminder of how their responses will be used /stored. |
